# Genomic features of pneumococcal strains isolated from paediatric patients with invasive disease during pneumococcal conjugate vaccine introduction in Lima, Peru

**DOI:** 10.1099/mgen.0.001621

**Published:** 2026-02-03

**Authors:** Brayan E. Gonzales, David Durand, Erik H. Mercado, Marcela Lopez-Briceño, Luis González, Grupo Peruano de Investigación en Neumococo (GPIN), Olguita Del Águila, Theresa J. Ochoa

**Affiliations:** 1 Instituto de Medicina Tropical Alexander von Humboldt, Universidad Peruana Cayetano Heredia, Lima, Peru; 2 Laboratorios de Investigación y Desarrollo (LID), Facultad de Ciencias e Ingeniería, Universidad Peruana Cayetano Heredia, Lima, Peru; 3 Department of Veterinary Medicine, University of Cambridge, Cambridge, UK; 4 Parasites & Microbes, Wellcome Sanger Institute, Hinxton, UK; 5 Facultad de Medicina, Universidad Peruana Cayetano Heredia, Lima, Peru

**Keywords:** antimicrobial resistance, clonal complexes, phylogenetic, pneumococcal conjugate vaccine, sequence types, whole-genome sequencing

## Abstract

To determine changes in the pneumococcal serotypes, sequence types (STs), clonal complexes (CCs) and the frequency of antimicrobial resistance genes after the introduction of pneumococcal conjugate vaccines (PCVs) in Lima, Peru. Retrospective multicentre study analysing whole-genome sequencing (WGS) data from three passive surveillance studies of invasive pneumococcal disease (IPD) in paediatric patients in Lima (2006–2020). Pneumococcal typing and antimicrobial resistance were analysed using *in silico* genomic tools. CCs were identified with eBURST and phylogenetic results were visualized using PHYLOViZ. 262 pneumococcal isolates were analysed (104 from IPD1, 70 from IPD2 and 88 from IPD3), 55.3% from children under 2 years old, 53.1% from patients with pneumonia and 28.5% with meningitis. After the introduction of PCVs, vaccine serotypes decreased, while serotype 19A and non-vaccine serotypes increased. The predominant STs were ST156 in IPD1 (*n*=25) and in IPD2 (*n*=7); and ST320 (*n*=38) and ST230 (*n*=15) in IPD3. Sixteen CC were identified, the most frequent were CC1421 (*n*=58) and CC156 (*n*=36). The overall penicillin non-susceptibility (NS) increased from 21.8% in IPD1 to 28.6% in IPD3, ceftriaxone-NS increased from 10% to 13.1% and macrolide-NS from 24.8% to 85.7% respectively. Resistance markers for macrolides, tetracycline and cotrimoxazole increased post-PCV13. WGS predicted antimicrobial resistance with high concordance, though some discrepancies were noted with phenotypic testing methods. Important changes in the distribution of serotype and ST, especially among vaccine serotypes, have been observed. These findings highlight the importance of monitoring vaccine effectiveness and tracking changes in bacterial populations to guide future vaccine implementation.

**Impact Statement**

This study used a collection of pneumococcal isolates causing invasive disease in Peru, covering more than 10 years during the sequential introduction of PCV7, PCV10 and PCV13. This is the only dataset providing a comprehensive view of pneumococcal evolution in Peru. We have contributed to understanding how key genomic features have changed following vaccine introduction in the region. The integration of whole-genome sequencing (WGS) into our pneumococcal surveillance studies has enabled the detection of changes beyond serotype alone, including shifts in sequence types (STs), and to compare the diversity of novel classifications such as Global Pneumococcal Sequence Cluster with conventional ST classifications. These insights show that the pneumococcal population continues to evolve in response to vaccine pressure. Continued genomic surveillance during the introduction of PCV15 and PCV20 in the region is essential to guide future vaccine development, implementation and empiric antibiotic treatment.

## Data Summary

Whole genome sequences have been deposited in the National Center for Biotechnology Information and the European Nucleotide Archive. IPD1 and IPD2 samples were included in BioProject PRJEB3084, while the last ten samples from IPD2 and the IPD3 samples were included in BioProject PRJNA830754. Four supplementary tables and the genomic metadata of the pneumococcal isolates have been submitted as a supplementary file. Requests for access to clinical and epidemiological data will be considered and approved upon review by the authors.

## Introduction

*Streptococcus pneumoniae* is a major cause of morbidity and mortality worldwide, especially in young children and older adults. *S. pneumoniae* is a common cause of upper respiratory infections (otitis, sinusitis) and more serious invasive infections such as pneumonia (with or without bacteraemia) and meningitis, known as invasive pneumococcal disease (IPD) [1]. In the Global Burden of Disease Study 2016, *S. pneumoniae* was the leading cause of lower respiratory infection morbidity and mortality globally (1.18 million deaths) [2]; and in 2019, it was the bacterial pathogen associated with the most deaths among children younger than 5 years [3].

The introduction of pneumococcal conjugate vaccines (PCVs) has reduced the incidence of invasive and non-invasive disease; however, changes in circulating serotypes and antibiotic resistance have occurred [4,7]. In general, vaccine serotypes have decreased and non-vaccine serotypes have increased; however, some particular serotypes, such as 3, 19A and 8, have risen in many regions [8]. Since serotype distribution varies by region and may be influenced by the vaccine used, the immunization schedule and vaccine coverage, it is essential to conduct epidemiological surveillance studies in different regions to monitor the impact of current vaccine strategies.

In Peru, the 7-valent pneumococcal conjugate vaccine (PCV7) was included in the national immunization programme in 2009, replaced by the 10-valent vaccine (PCV10) in 2011 and by the 13-valent vaccine (PCV13) in June 2015. In 2018, PCV13 was also introduced for immunization of adults over 60 years of age. To monitor changes in serotypes and antibiotic resistance, our research group – Peruvian Pneumococcus Research Group/Grupo Peruano de Investigación en Neumococo (GPIN) – has conducted three passive surveillance IPD studies in Lima, including all isolates causing IPD, independent of serotype. The first study (IPD1) was conducted between 2006 and 2008 before PCV7 introduction [9]; the second (IPD2) was conducted between 2009 and 2011 after PCV7 but before PCV10 introduction [10]; and the third study (IPD3), between 2016 and 2019, following the introduction of PCV13 in children [11].

We conducted this study to describe the genomic features of pneumococcal strains, based on whole-genome sequencing (WGS) analysis of isolates from our previous three studies in paediatric patients with invasive disease, both before and after the introduction of PCV in Peru. The aims of this study were [1] to determine the changes in the distribution of serotypes, sequence types (STs) and clonal complexes (CCs) of *S. pneumoniae* over time [2], to assess phylogenetic diversity among invasive isolates and [3] to determine the relationship between genotypic predictors of antimicrobial resistance (AMR) and phenotypic antimicrobial susceptibility.

## Methods

### Study design, population and sample collection

This was a cross-sectional, retrospective and multicentre study. We performed a secondary data analysis of three passive surveillance studies of IPD conducted by the GPIN. *S. pneumoniae* cultures from normally sterile sites were collected from patients hospitalized in private and public hospitals in Lima, Peru. The first study (IPD1), conducted between 2006 and 2008 prior to the introduction of PCV7 (pre-PCV7), included patients under 16 years of age [9]; IPD2, between 2009 and 2011, after the introduction of PCV7 (post-PCV7), included paediatric and adult patients [10]; and IPD3, between 2016 and 2019, after the introduction of PCV13 (post-PCV13), included paediatric and adult patients [11]. All studies had the same enrolment criteria, except for the differences in age. For the current study, we included only *S. pneumoniae* strains from patients <18 years old.

### Laboratory studies

*S. pneumoniae* strains collected in the previous studies, stored at −80 °C in skim milk-tryptone-glucose-glycerol medium, were reconstituted using blood agar plates, Gram stain, optoquine and bile solubility tests were performed to reconfirm the pneumococcal strains. Pure colonies were sent to the Streptococcus Lab of the US Centers for Disease Control and Prevention (CDC) for WGS in collaboration with the Global Pneumococcal Sequencing Project (GPS) at the Wellcome Sanger Institute.

### Molecular typing and antibiotic resistance predictors

The genomic analyses were performed using the GPS pipeline, which includes a series of bioinformatics tools [12]. The pneumococcal typing pipeline (SeroBA) was used to identify *in silico* serotypes, multilocus sequence typing (MLST) and pilus genes. Allelic numbering, allelic profile of seven genes and STs were assigned using software available at the *S. pneumoniae* MLST database web page (http://pubmlst.org/spneumoniae). Global Pneumococcal Sequence Cluster (GPSC), a novel genomic definition of pneumococcal lineage, was defined using PopPUNK [13] in order to compare genetic diversity with more conventional classification (serotype and ST).

Molecular mechanism of antibiotic resistance to beta-lactam resistance was inferred from penicillin-binding proteins (PBPs) genes 1 a, 2b and 2x; chloramphenicol resistance was inferred from *cat* gene; macrolide resistance involved *erm*B, *mef*A and *msr*D genes, as well as mutations in 23S rRNA (R23S1) and ribosomal proteins (RPLD2); trimethoprim-sulfamethoxazole (co-trimoxazole) resistance was linked to mutations in *fol*A and *fol*P; tetracycline resistance was detected via the *tet*M gene; fluoroquinolone resistance was predicted based on substitutions in the *gyr*A and *par*C genes. Additional resistance determinants, including rare substitutions and mobile genetic elements (e.g. cassettes or plasmids), were also identified [*dfr*16, *rpo*B1, aph(3′), *bla*TEM and *qep*A] and analysed from the WGS data as mentioned in previous studies using the CDC PBP AMR Predictor pipeline and ARIBA v2.14.6 [14]. The discrepancies between the antimicrobial resistance predicted by WGS and the phenotypic resistance evaluated by minimum inhibitory concentration (MIC; determined by E-test) or Kirby-Bauer method (KB; disc diffusion test) (phenotypic data collected from the previous publications) were categorized as minor discrepancies (intermediate by phenotypic methods but inferred as susceptible by WGS or susceptible by phenotype but inferred intermediate by WGS), major discrepancies (susceptible by phenotypic methods but inferred as resistant by WGS) and very major discrepancies (resistant by phenotypic methods but inferred as susceptible by WGS) based on the paper from Gagetti *et al*. [15], based on its clinical relevance for antibiotic management.

### Clonal complex (CC) determination and phylogenetic relationships

Analysis of CCs was performed using all STs found in the online database using the eBURST program. STs were grouped into CCs by their similarity to one or more central allelic profiles (core STs), and the CC name is assigned according to the core STs. Visualization of phylogenetic results was performed using the PHYLOViZ online tool (http://www.phyloviz.net/). Each ST is related to other STs by the number of allelic differences: single-locus variant, double-locus variant and triple-locus variant. The phylogenetic relationship of serotype 19A was evaluated from MLST through Unweight pair group method using Arithmetic averages (UPGMA) analysis, which consists of evaluating the most similar STs and calculating the average distances between them.

### Statistical analysis

The frequency of virulence factors and antibiotic resistance genes between isolates from IPD studies was compared using the chi-square test (*χ*^2^) and Fisher’s exact test. Diversity in serotype, ST and GPSC was evaluated with Simpson’s diversity index and 95% confidence intervals from 1,000 bootstrap replicates. These analyses were conducted using Stata/SE v.19.0 and R v4.5.0. The statistical significance level was set at *P*<0.05.

### Ethical aspects

This study was approved by the Institutional Review Board of Universidad Peruana Cayetano Heredia (Lima, Peru) and by the ethics committees of each hospital participating in the previous IPD studies.

## Results

A total of 262 pneumococcal isolates from IPD in paediatric patients with WGS data were included in this study; 104 from IPD1, 70 from IPD2 and 88 from IPD3 (including 14 additional strains that were not part of the original publications but were collected using the same inclusion criteria during each period, 10 in IPD2 and 4 in IPD3) [9,11] (Supplementary Material: Supplementary_Metadata.xlsx). Of these isolates, 54.7% were from male patients, 55.3% from infants under 2 years of age, 31.0% were collected during the winter season in Peru (21 June to 23 September), and 59.1% were isolated from blood cultures. Pneumonia was the primary diagnosis (53.1%), followed by meningitis (28.5%). The age of the children, the primary clinical diagnosis and the discharged status significantly changed over time. We had discharge information on 215 patients; of these, 30 died, with an overall case fatality rate of 14.0%. The case fatality rate was higher during pre-PCV7 (22.0%; 95% CI: 14.6–31.7%) compared with post-PCV7 (7.4%; 95% CI: 2.8–18.2%) and post-PCV13 (8.6%; 95% CI: 3.9–17.9%) (*P*=0.003). However, since the discharge status was not available for all patients, this may overestimate these percentages (Table 1).

**Table 1.** Characteristics of study population with invasive pneumococcal disease (*N*=262)*

Characteristic	Total *N*=**262** n (%)	Study			*P* ¶
		IPD1	IPD2	IPD3	
		*N*=**104**	*N*=**70**	*N*=**88**	
		n (%)	n (%)	n (%)	
Sex					0.427
Female	116 (45.3)	41 (40.6)	32 (46.4)	43 (50.0)	
Age group (years)					<0.001
Infants (<2)	145 (55.3)	74 (71.2)	31 (44.3)	40 (45.5)	
Pre-school children (≥2-<6)	66 (25.2)	17 (16.4)	17 (24.3)	32 (36.4)	
School children (≥6-<18)	51 (19.5)	13 (12.5)	22 (31.4)	16 (18.2)	
Seasonal distribution					0.795
Winter	81 (31.0)	32 (30.8)	19 (27.5)	30 (34.1)	
Fall	74 (28.4)	29 (27.9)	21 (30.4)	24 (27.3)	
Spring	61 (23.4)	21 (20.2)	18 (26.1)	22 (25.0)	
Summer	45 (17.2)	22 (21.2)	11 (15.9)	12 (13.6)	
Culture site					0.002
Blood	153 (59.1)	59 (56.7)	42 (61.8)	52 (59.8)	
Cerebrospinal fluid	52 (20.1)	30 (28.9)	13 (19.1)	9 (10.3)	
Pleural fluid	40 (15.4)	9 (8.7)	7 (10.3)	24 (27.6)	
Others †	14 (5.1)	6 (5.8)	6 (8.8)	2 (2.4)	
Primary clinical diagnosis					0.045
Pneumonia	138 (53.1)	52 (50.0)	34 (50.0)	52 (59.1)	
Meningitis	74 (28.5)	41 (39.4)	17 (25.0)	16 (18.2)	
Bacteremia‡	35 (13.5)	7 (6.7)	12 (17.7)	16 (18.2)	
Others §	13 (5.1)	4 (3.9)	5 (7.4)	4 (4.5)	
Discharge status					0.003
Dead/CFR**	30/215 (14.0)	20/91 (22.0)	4/54 (7.4)	6/70 (8.6)	
Live with sequelae	49 (22.8)	11 (12.1)	18 (33.3)	20 (28.6)	
Live without sequelae	136 (63.3)	60 (65.9)	32 (59.3)	44 (62.9)	

*Some variables may add less than 262 due to missing data.

†Peritoneal fluid, ovarian abscess, skin abscess, bronchial aspirate, bile, tracheal aspirate and joint fluid.

‡Without pneumonia or meningitis.

§Bacterial peritonitis, septic arthritis, colitis and fasciitis.

¶Chi-square test (χ2) (comparison across the three study periods)

**CFR, case fatality rate

IPD, Invasive pneumococcal disease.

### Change in the distribution of serotypes and sequence types

**Fig. 1.**
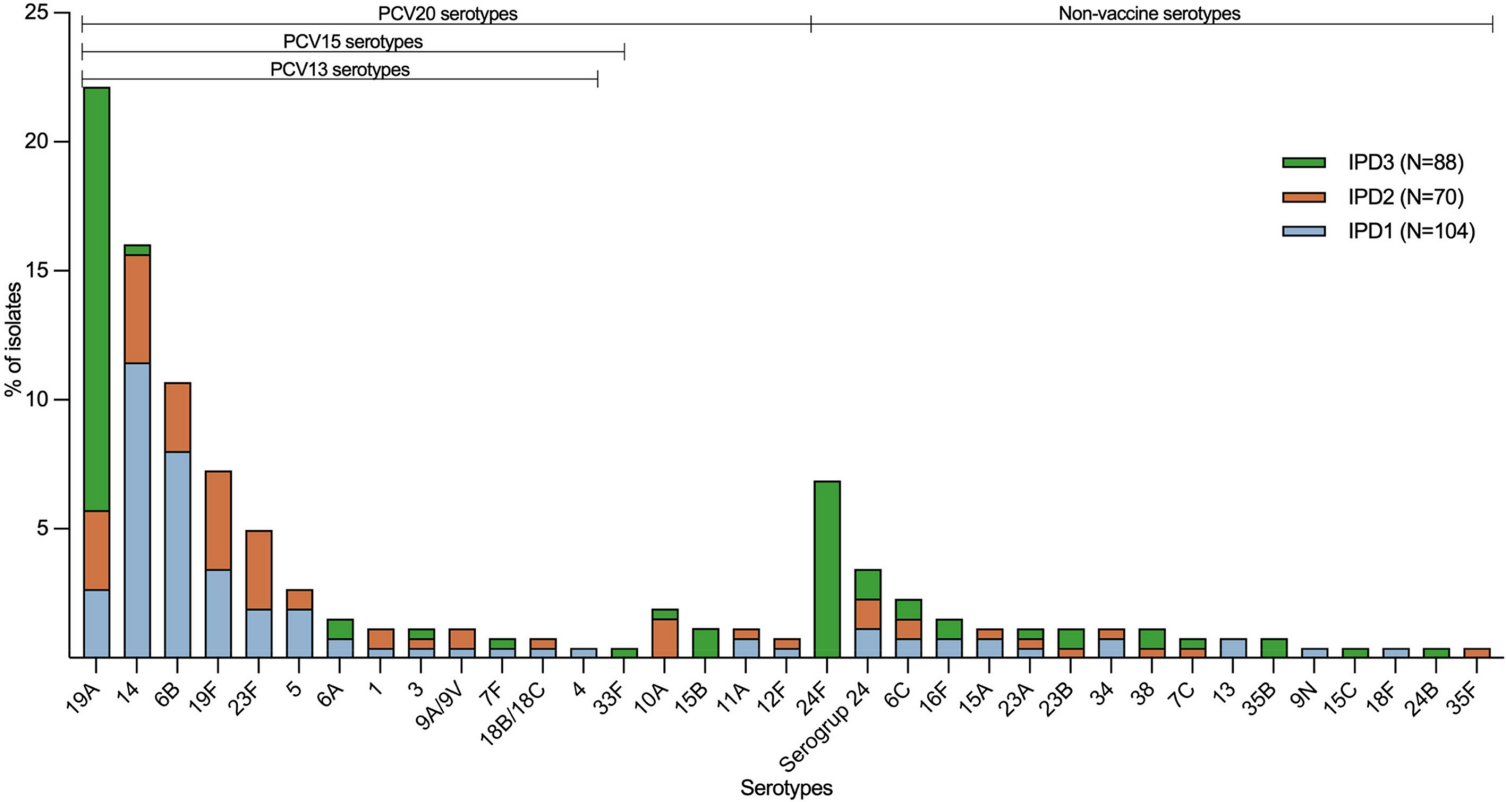
Distribution of pneumococcal serotypes isolated from children with invasive pneumococcal disease in Lima, Peru, from 2009 to 2019 (*N*=262). The serotypes are displayed in order of frequency for the total number of strains across all three studies (IPD1=pre-PCV7, IPD2=post-PCV7, and IPD3=post-PCV13), starting with the PCV13 serotypes, followed by the additional PCV15 and PCV20 serotypes, and finally the non-PCV20 vaccine serotypes.

The distribution of serotypes changed over time, particularly in vaccine serotypes (Fig. 1). Serotypes 14, 6B, 19F and 23F showed a substantial reduction after the introduction of PCV7 and PCV10. Serotype 14 decreased from 28.9% to 15.7% to 1.1% in IPD1, IPD2 and IPD3, respectively; similarly, serotype 6B decreased from 20.2% to 10.0% to 0%, respectively. However, serotype 19A (PCV13 vaccine-type) emerged as the predominant serotype right after PCV13 introduction (IPD3); it increased from 6.7% in IPD1 to 11.4–48.9% in IPD3. Furthermore, non-vaccine serotypes such as 24F, 6C and 16F appear to be emerging. Serotype 24F was absent in the first two studies, but in IPD3, it represented 20.5% of isolates (Fig. 1).

The serotype and ST distribution according to study period are presented in Table 2. Before PCV7 introduction (IPD1), ST156 was the predominant ST (24%) (25 strains, 23 of them belonging to serotype 14), followed by ST1421 (5.8%) (6 strains, all 19F) and ST289 (4.8%) (5 strains, all serotype 5). After PCV7 introduction (IPD2), ST156 was the predominant (10%) (7 strains, all serotype 14), ST1421 (7.1%) (5 strains, all 19F) and ST242 (7.1%) (5 strains, all 23F). Right after the introduction of PCV13 (IPD3), ST320 became the predominant strain (43.2%) (38 strains, all 19A), followed by ST230 (17%) (15 strains, 13 of which were 24F).

**Table 2.** Serotype and ST distribution of pneumococcal strain isolated from children with IPD according to vaccine serotype and study period during PCVs introduction in Peru (*N*=262)

WGS serotype	ST	N	Study		
			IPD1 (*n*=104)	IPD2 (*n*=70)	IPD3 (*n*=88)
			(Pre-PCV7)	(Post-PCV7)	(Post-PCV13)
4	206	1	1		
6B	90	4	2	2	
	135	3	2	1	
	315	1	1		
	902	1	1		
	1121	3	3		
	1292	1		1	
	1624	1	1		
	1662	1	1		
	5449	4	3	1	
	5450	1	1		
	5619	1	1		
	5625	6	4	2	
	5626	1	1		
9A/9V	156	1	1		
	280	2		2	
14	15	3	3		
	25	2	2		
	156	31	23	7	1
	646	1	1		
	5458	1		1	
	6144	1		1	
	7432	1		1	
	9054	1		1	
	9912	1	1		
18B/18C	5451	1	1		
	9925	1		1	
19F	81	2	2		
	646	1	1		
	1203	1		1	
	1421	11	6	5	
	5459	1		1	
	7132	1		1	
	9904	1		1	
	newST_1	1		1	
23F	81	4	1	3	
	156	1	1		
	242	8	3	5	
1	615	3	1	2	
5	289	7	5	2	
7F	191	1			1
	5455	1	1		
3	180	1			1
	5616	1	1		
	9060	1		1	
6A	273	1	1		
	1876	1			1
	5623	1	1		
	9463	1			1
19A	66	1	1		
	276	4	3		1
	320	42	2	2	38
	1131	3		2	1
	1451	2			2
	2013	1		1	
	5452	1	1		
	5460	1		1	
	6048	1		1	
	newST_3	1		1	
	newST_c	1			1
33F	1012	1			1
10A	5472	3		3	
	9055	1		1	
	18188	1			1
11A	62	1		1	
	193	1	1		
	4063	1	1		
12F	218	2	1	1	
15B	3066	1			1
	3557	1			1
	7479	1			1
6C	1292	6	2	2	2
7C	5468	2		1	1
9 N	66	1	1		
13	5593	2	2		
15A	5448	2	1	1	
	5453	1	1		
15C	5461	1			1
16F	5673	1	1		
	6149	1	1		
	7438	2			2
18F	5456	1	1		
23A	338	1			1
	439	2	1	1	
23B	1349	2			2
	6140	1		1	
Serogroup 24	230	4		3	1
	5033	2			2
	5581	1	1		
	6139	2	2		
24B	230	1			1
24F	230	13			13
	338	1			1
	4253	1			1
	18195	1			1
	18232	1			1
	newST_k	1			1
34	1902	1		1	
	5447	1	1		
	7441	1	1		
35B	18160	1			1
	18270	1			1
35F	5600	1		1	
38	5475	2		1	1
	18244	1			1

Serotypes according to PCVs: PCV7 (Orange). Additional serotypes included in PCV10 (Yellow). Additional serotypes included in PCV13 (Green). Additional serotypes included in PCV15 (Blue). Additional serotypes included in PCV20 (Grey).

IPD, Invasive pneumococcal disease; newST, new sequence type; NT, Non-typeable strain by WGS.

In the post-PCV13 period (IPD3), among 43 isolates of serotype 19A, 76% (38/43) were identified as ST320. In addition, four new STs were identified post-PCV7; two strains had each allele identified, but the ST was not assigned (newST_letter), and two strains had some of the alleles not identified (newST_number) (Table 2). Since serotype 19A was the predominant serotype in the third period, we wanted to measure how diverse the overall distribution of strains was as a result of vaccine introduction. The IPD3 study revealed a lower diversity of serotypes, STs and GPSCs, as indicated by Simpson’s index, compared to the two previous studies (Table 3). Additionally, the diversity analysis showed that regardless of whether diversity was assessed using GPSC, serotypes or ST, IPD3 consistently showed lower diversity, suggesting that in our population, GPSC-based classification provides a level of diversity comparable to conventional classification.

**Table 3.** Diversity of pneumococcal serotypes, ST and GPSC by study

Study	Simpson’s diversity (95% CI)		
	Serotype	ST	GPSC
IPD study			
IPD1	0.86 (0.78–0.94)	0.92 (0.89–0.98) *	0.90 (0.86–0.95)
IPD2	0.91 (0.88–0.93)	0.96 (0.95–0.97)	0.95 (0.92–0.96)
IPD3	0.71 (0.57–0.94)	0.78 (0.68–0.97)	0.73 (0.58–0.95)

The Simpson’s diversity was compared across studies (IPD1 vs IPD2, IPD2 vs IPD3 and IPD1 vs IPD3) for serotype, ST and GPSC using the Wilcoxon rank test. The only significant difference * (*P*=0.024) was for the comparison of ST between IPD1 and IPD3.

95%CI: was estimated using 1000 bootstrap values.

Close to 1: Diversity.

Close to 0: Predominant (no diversity).

GPSC, Global Pneumococcal Sequencing Cluster; IPD, Invasive pneumococcal disease.

### Phylogenetic relationships

**Fig. 2.**
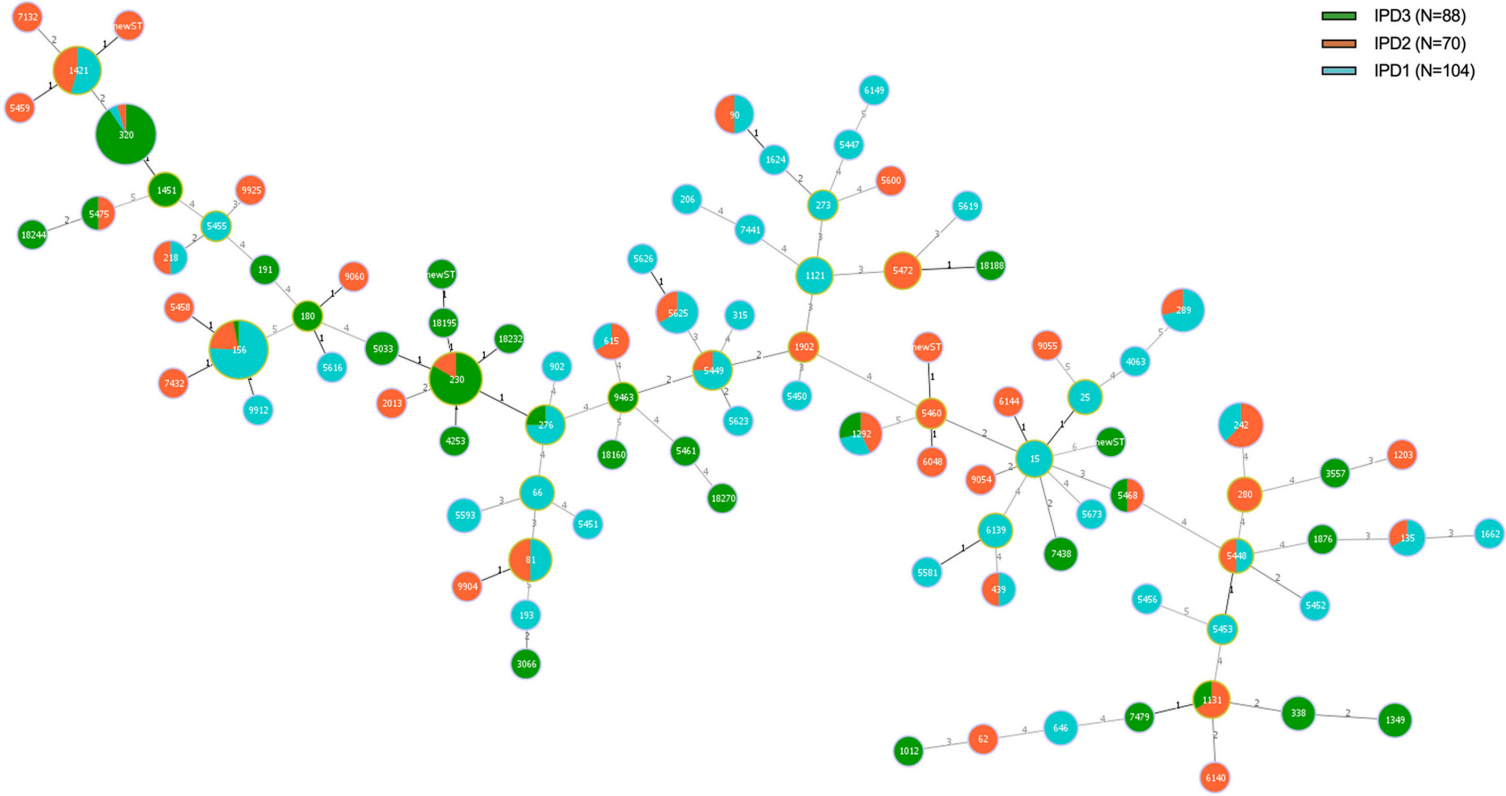
Distribution of ST in pneumococcal isolates from patients with IPD (*N*=262). Note: the figure shows the distribution of 93 ST identified by MLST. ST size was proportional to the number of isolates. Each colour represents isolates from the three studies. There are multiple branches, considering that the allelic relationship was set up for two alleles.

**Fig. 3.**
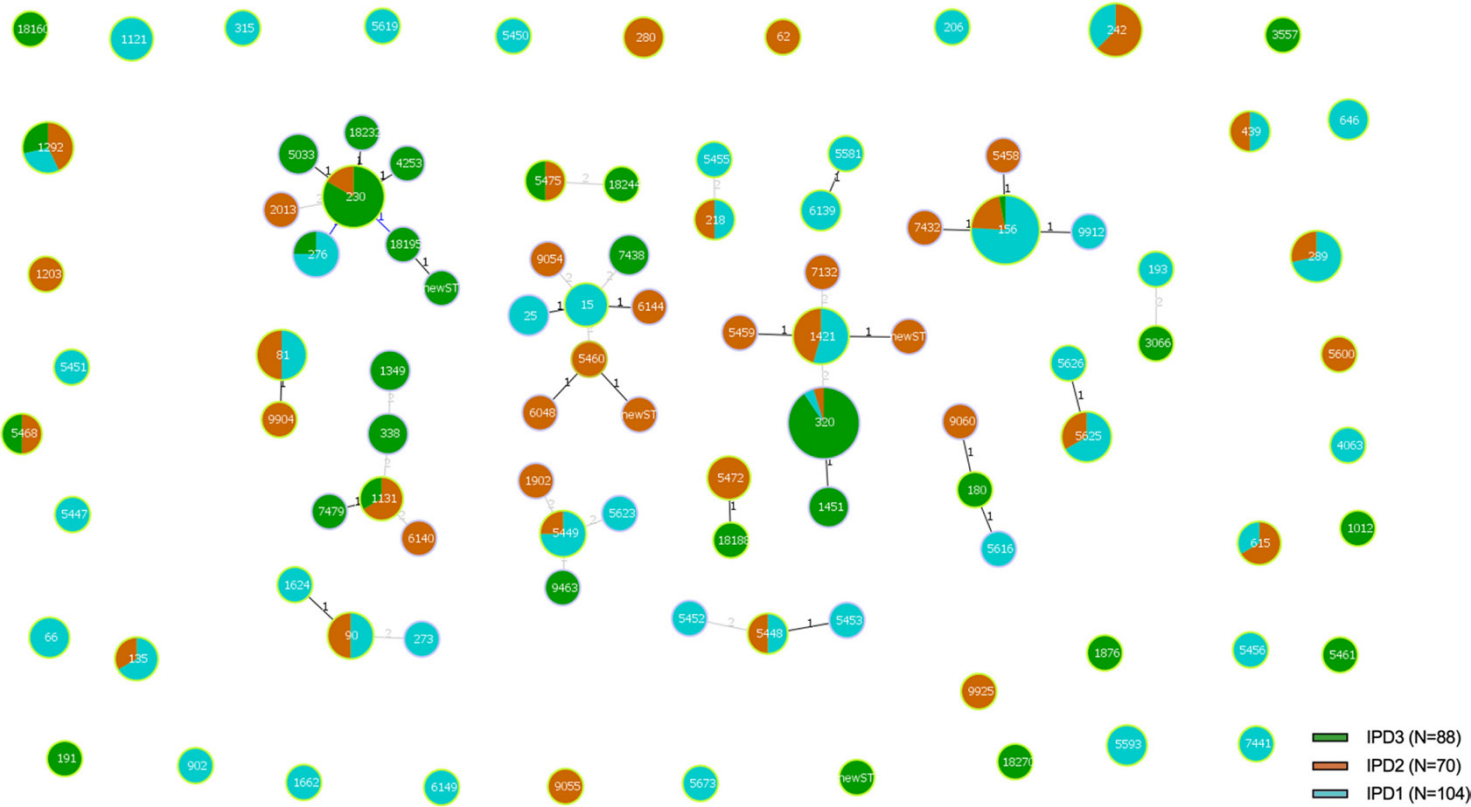
Distribution of CCs in pneumococcal isolates from patients with IPD (*N*=262). Note: the figure shows 16 CCs and 38 singletons. CC size was proportional to the number of isolates. CC1421 (including ST320 isolates), CC156, CC230 and CC5460/15 were the main CC. Each colour represents isolates from the three studies.

The PHYLOVIZ analysis based on MLST alleles showed a diverse population structure with no clear clustering by study period (Fig. 2). To assess the phylogenetic relationships, our analysis identified 16 CCs and 38 singleton STs (Fig. 3). The most prevalent CCs were CC1421 (*n*=58, which includes the dominant ST320), CC156 (*n*=36), CC230 (*n*=29) and CC5460/15 (*n*=12), which together accounted for 51.5% of all isolates.

### Antimicrobial resistance

Of the 262 strains, phenotypic antimicrobial resistance was performed in 248 (94.7%). The overall penicillin non-susceptibility (NS) increased from 21.8% in IPD1 to 28.6% in IPD3, ceftriaxone-NS increased from 10% to 13.1% and macrolide-NS from 24.8% to 85.7% respectively. The changes in resistance over time for all antibiotics are presented in Table 4.

**Table 4.** Changes in antimicrobial resistance of pneumococcal strain isolates from children with IPD during PCV introduction in Peru (*n*=248)

Antibiotic *	Non-susceptible (Resistant/Intermediate)		
	IPD1 (*n*=104)	IPD2 (*n*=60)	IPD3 (*n*=84)
	(Pre-PCV7)	(Post-PCV7)	(Post-PCV13)
	n (%)	n (%)	n (%)
Penicillin †	22 (21.8)	7 (12.5)	24 (28.6)
Ceftriaxone †	10 (10.0)	8 (14.3)	11 (13.1)
Macrolides ‡	25 (24.8)	22 (39.3)	72 (85.7)
Tetracycline	48 (46.2)	40 (67.8)	61 (72.6)
Clindamycin	15 (14.4)	10 (16.7)	59 (70.2)
Chloramphenicol	12 (11.9)	6 (10.9)	18 (21.4)
SXT	76 (73.1)	37 (61.7)	69 (82.1)

*Some antibiotics may add less than the total per study due to some isolates not being recovered for antibiotic susceptibility testing.

†Include the interpretation of meningitis and non-meningitis breakpoints according to the patient’s diagnosis.

‡Erythromycin and azithromycin.

IPD, Invasive pneumococcal disease; SXT, trimethoprim-sulfamethoxazole.

### Virulence factors and antibiotic resistance markers

To evaluate gene composition beyond serotypes, we examined other important traits such as virulence factors and resistance markers. Overall, we found a 23.3% prevalence of variant pathogenicity islet 1 (PI-1) and the exact prevalence for PI-1 and PI-2 present at the same time (PI-1+2). The prevalence of isolates with only PI-1 decreased significantly from 40.4% in IPD1 to 6.8% in IPD3 (*P*<0.001), whereas the prevalence of PI-1+2 increased significantly from 7.7% in IPD1 to 45.5% in IPD3 (*P*<0.001) (Table 5). About antibiotic resistance genes, the presence of *erm*B or *mef*A increased from 25% (both genes negative 75%) in IPD1 to 77.3% (both genes negative 22.7%) in IPD3 (*P*<0.001) and the frequency of *msr*D (associated with macrolide and streptogramin B resistance) increased from 13.5% to 50% (*P*<0.001). Overall, the *tet*M gene (recognized as conferring tetracycline resistance) was detected in 46.2%; and both *fol*A+*fol*P mutations (associated with cotrimoxazole resistance) were detected in 63.7% of strains. Among all analysed isolates, a sample from IPD1 had 12 resistance markers [*erm*B+*mef*A+* ms*rD + *tet*M+* cat + fol*A+* fo*lP + *gyr*A (S11F) + *par*C (S2Y) + aph(3′)-lll (plasmid)+ant (6) + *sat*4] and a sample from IPD2 had 11 resistance markers [*erm*B+*mef*A+* ms*rD + *tet*M+* fo*lA + *fol*P+* gy*rA (S11F) + *par*C (S2Y) + aph(3′)-III (plasmid)+ant [6] + *sat*4] (Table 5).

**Table 5.** Virulence factor and predictors of antibiotic resistance by gene expression profiles of pneumococcal isolates (*N*=262)

Characteristic	Total *N*=**262** n (%)	Study			*P* *
		IPD1	IPD2	IPD3	
		*N*=**104**	*N*=**70**	*N*=**88**	
		n (%)	n (%)	n (%)	
Pilus					<0.001
Negative	136 (51.9)	53 (51.0)	42 (60.0)	41 (46.6)	
PI-1	61 (23.3)	42 (40.4)	13 (18.6)	6 (6.8)	
PI-2	4 (1.5)	1 (1.0)	2 (2.9)	1 (1.1)	
PI-1+2	61 (23.3)	8 (7.7)	13 (18.6)	40 (45.5)	
Macrolide					<0.001
Negative	137 (52.3)	78 (75.0)	39 (55.7)	20 (22.7)	
*erm*B+*mef*A	62 (23.7)	8 (7.7)	14 (20.0)	40 (45.5)	
Only *erm*B	42 (16.0)	12 (11.5)	6 (8.6)	24 (27.3)	
Only *mef*A	21 (8.0)	6 (5.8)	11 (15.7)	4 (4.6)	
Macrolide and streptogramin B					<0.001
Negative	179 (68.3)	90 (86.5)	45 (64.3)	44 (50.0)	
*msr*D	83 (31.7)	14 (13.5)	25 (35.7)	44 (50.0)	
Tetracycline					<0.001
Negative	141 (53.8)	78 (75.0)	40 (57.1)	23 (26.1)	
*tet*M	121 (46.2)	26 (25.0)	30 (42.9)	65 (73.9)	
Chloramphenicol					0.009 †
Negative	249 (95.0)	97 (93.3)	64 (91.4)	88 (100.0)	
*Cat*	13 (5.0)	7 (6.7)	6 (8.6)	0	
Cotrimoxazole					0.060 †
Negative	50 (19.1)	21 (20.2)	18 (25.7)	11 (12.5)	
*fol*A+*fol*P	167 (63.7)	70 (67.3)	43 (61.4)	54 (61.4)	
Only *fol*A	1 (0.4)	0	0	1 (1.1)	
Only *fol*P	44 (16.8)	13 (12.5)	9 (12.9)	22 (25.0)	
Fluoroquinolone					0.732 †
Negative	2 (0.8)	1 (1.0)	1 (1.4)	0	
*gyr*A+*par*C	260 (99.2)	103 (99.0)	69 (98.6)	88 (100.0)	
Rare plasmid					
aph(3′) ‡	3 (100.0)	2 (100.0)	1 (100.0)	0	

*Chi-square test (χ2) (comparison across the three study periods)

†Fisher’s exact (comparison across the three study periods)

‡Plasmid confers aminoglycoside resistance.

Note: strains with greater antimicrobial resistance markers:

A sample from IPD1 had 12 resistance markers →* erm*B* + mef*A* + msr*D* + tet*M* + cat + fol*A* + fol*P* + gyr*A (S11F) *+ par*C (S2Y)* + *aph(3′)-lll (plasmid) + ant(6) + sat4

A sample from IPD2 had 11 resistance markers →* ermB*B+*mef*A+* ms*rD + *tet*M+* fo*lA + *fol*P+* gy*rA (S11F) + *par*C (S2Y) + aph(3′)-III (plasmid)+ant(6) + sat4

IPD, Invasive pneumococcal disease.

### Comparison between WGS resistance prediction and phenotypic resistance

The antimicrobial resistance of penicillin, ceftriaxone, macrolides and tetracycline was predicted by WGS and evaluated by MIC or KB. Discrepancies were categorized as minor, major and very major discrepancies (Table 6). A total of 241 isolates were evaluated for resistance to penicillin, ceftriaxone and macrolides, yielding concordance rates of 96.6%, 70.1% and 92.1%, respectively (Table 6). Only one isolate showed a very major discrepancy (resistant by phenotypic methods but susceptible as predicted by WGS) for penicillin and ceftriaxone, while five isolates showed a very major discrepancy in macrolide resistance. Additionally, among 247 isolates evaluated for predicted tetracycline resistance, a concordance of 78.1% was found and very major discrepancies in 7 isolates. Other discrepancy patterns (different from those shown in Table 6) were found in 33 isolates for macrolide susceptibility and 10 isolates for tetracycline susceptibility. Detailed descriptions of inconsistencies between the antibiotic resistance predictors derived from WGS and the antibiotic resistance assays, including information on the serotype, ST and GPSC, are provided in Materials S1-S4, available in the online version of this article.

**Table 6.** Agreement between the phenotype and genotype (WGS) antimicrobial resistance of pneumococcal isolates to four antibiotics

Antibiotic	No. of isolates	Phenotypic resistance method	Concordance (%)	Discordance with WGS			
				Minor discrepancy § n (%)	Major discrepancy ¶ n (%)	Very major discrepancy ** n (%)	Others †† n (%)
Penicillin *	241	MIC by E-test	233 (96.6)	–	7 (2.9)	1 (0.5)	–
Ceftriaxone †	241	MIC by E-test	169 (70.1)	37 (15.4)	10 (4.1)	1 (0.5)	33 (9.9)
Macrolide ‡	241	MIC by E-test	222 (92.1)	8 (3.3)	6 (2.5)	5 (2.1)	–
Tetracycline	247	Kirby Bauer	193 (78.1)	32 (13.0)	5 (2.0)	7 (2.8)	10 (4.1)

*Meningitis breakpoint. S: MIC≤0.06 ug ml−1, R: MIC≥0.12 ug ml−1.

†Meningitis breakpoints. S MIC≤0.5 ug ml−1, I: MIC=1 ug ml−1, R: MIC≥2 ug ml−1.

‡Isolates included in IPD1 and IPD2 were evaluated to erythromycin and IPD3 were evaluated to azithromycin.

§Intermediate by phenotypic methods but susceptible predicted by WGS or susceptible by phenotypic methods but intermediate predicted by WGS.

¶Susceptible by phenotypic methods but resistant predicted by WGS.

**Resistant by phenotypic methods but susceptible predicted by WGS.

††Others discordance Intermediate by phenotypic methods but resistant predicted by WGS/resistant by phenotypic methods but intermediate predicted by WGS.

### Phylogenetic relationships among serotype 19A isolated from IPD patients

**Fig. 4.**
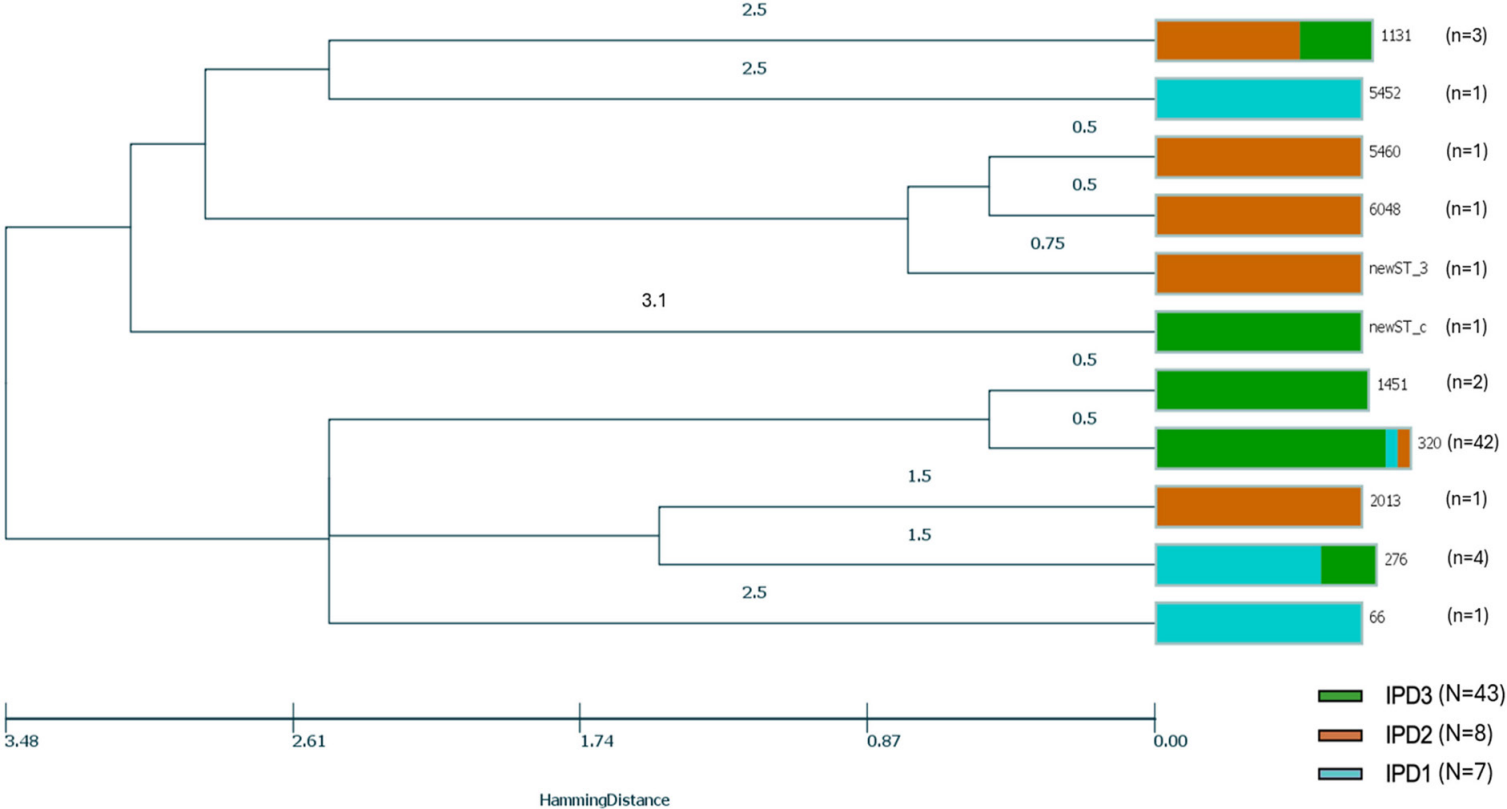
Phylogenetic relationships among *S. pneumoniae* serotype 19A from children with IPD (*N*=58). Note: UPGMA analysis shows a specific cluster among 19A isolates formed by ST320, representing 76%(44/58) of the isolates. The size of the colour was proportional to the number of isolates among the same ST. Each colour represents isolates from the three studies.

We performed a phylogenetic analysis of serotype 19A to investigate whether the observed increase in its frequency over time was associated with the emergence or expansion of specific clones. Serotype 19A was the most common serotype overall (58/262 strains, 22.1%) and the predominant one in IPD3 (43/88, 48.9%). The phylogenetic relationship of serotype 19A was evaluated using MLST, followed by UPGMA analysis. The formation of two internal nodes was observed. The first node was identified as a clade among isolates from IPD2 with ST5460, ST6048 and newST_3. The second node was a clade related mainly to IPD3 with ST320 and ST1451, the latter representing 72.4% (*n*=42) of the sequences (Fig. 4).

## Discussion

This study describes the changes in pneumococcal serotypes, STs and CCs, as determined by WGS, following the introduction of PCVs in Lima, Peru. While PCV7 vaccine serotypes (14, 6B, 19F and 23F) decreased substantially, serotype 19A (PCV13 vaccine type) emerged as the predominant serotype in Peru, representing almost half of all invasive isolates, followed by serotype 24F. Similarly, the distribution of ST has changed accordingly. The predominant STs before PCV introduction were ST156 (mainly serotype 14), ST1421 (serotype 19F) and ST289 (serotype 5). Following the introduction of PCV13, ST320 (serotype 19A) became the predominant ST, followed by ST230 (mainly serotype 24F). In our collection of strains, four CCs accounted for half of all strains; the most predominant were CC1421 (including ST320), CC156 and CC230. In general, during the post-PCV13 period, we observed a decrease in the diversity of serotypes, ST and GPSC compared to previous periods; however, we found higher rates of antimicrobial resistance. We also found a significant decrease in the case fatality rate of our IPD cases, highlighting the overall positive impact of PCV introduction, as described in many countries [1,4,7].

The most striking finding is the predominance of serotype 19A following the introduction of PCV13. Although this could be related to incomplete vaccination and low population vaccination coverage immediately after the vaccine was introduced (our IPD3 study was conducted 16 months after the start of PCV13 vaccination in Peru), it could also represent vaccine failure or vaccine escape. In our previous study, where we described in more detail the yearly distribution of serotype 19A after PCV13 introduction, we observed a decrease in 19A IPD cases in the last year of the study, although it was not statistically significant due to the small number of strains [11]. Nevertheless, since long-term follow-up data are not yet available, we cannot determine whether this represents an early, temporary replacement event that might stabilize or decline with higher PCV13 coverage or if it indicates a more persistent niche for this serotype. In the USA and many other countries after PCV7 introduction, the predominant serotype became 19A, due mainly to selective pressure and in part because of vaccine escape [7,16,18]. Post-PCV7, 19A-ST320 became a highly prevalent multidrug-resistant genotype in carriage and IPD studies [7]. In addition, in countries that used PCV10, the frequency of serotype 19A increased, accompanied by a selection of CC320 (which includes ST320) and antimicrobial resistance [19]. In the recent WHO-commissioned Pneumococcal Serotype Replacement and Distribution Estimation (PSERENADE) project, serotype distribution differed between countries using PCV10 or PCV13; in PCV10 countries, serotype 19A was by far the most common (30.6%), followed by serotype 3 (8.4%) in children younger than 5 years old [20]. Thus, the previous use of PCV10 in Peru, prior to the switch to PCV13, may have contributed to the higher prevalence of serotype 19A in Lima. On the other hand, after the introduction of PCV13, specific subclades of serotype 19A have been associated with vaccine failures, with GPSC1-CC320 being the most prevalent [21]. All these factors could explain why serotype 19A has not been entirely eradicated and remains responsible for an important percentage of IPD cases globally.

In our study, after PCV13 introduction, the most prevalent STs were ST320 (33%) and ST230 (17%); 76% of serotype 19A isolates were ST320. These findings are comparable to those reported in some countries in the region. For instance, in a paediatric study conducted in Colombia in 2016, 29.4% of pneumococcal isolates were serotype 19A, and 80% of these 19A isolates belonged to ST320 [22]. Likewise, in Chile in 2015, 48% meningitis cases by serotype 19A were ST320 [23]. While in Argentina, during the PCV13 era, the predominant ST was ST306 (associated with serotype 1), and among 12 serotype 19A isolates, only one was ST320 [15]. Moreover, CC/ST320 and CC/ST230 did not exclusively emerge after PCV introduction in specific regions. For instance, in Poland (2010–2016), before the introduction of PCV, both STs were predominant in IPD cases in infants [24]. Thus, while similar trends are observed in some countries, the distribution of ST320 associated with serotype 19A varies geographically and is not necessarily related to vaccine-selective pressure. This highlights the importance of genomic surveillance to identify emerging serotypes/STs/CCs that new PCVs may potentially cover.

Two years after the introduction of PCV13 in Peru, serotype 24F, a non-vaccine serotype, has emerged as the second most frequent serotype, following serotype 19A. This phenomenon has been described in several countries following the introduction of PCV13 [15,25]. This serotype has been associated with multidrug resistance and potential invasiveness, necessitating ongoing monitoring [25]. In a sub-analysis of the pneumonia cases in our series, we found that serotype 24F was not present in the first two periods (pre-PCV13). Still, during the post-PCV13 period, it accounted for 22% of all pneumococcal pneumonia cases in Lima [26].

A lower pneumococcal serotype diversity was observed in IPD3 compared with IPD2 and IPD1 (based on the Simpson’s index), mainly because sufficient time had elapsed between the first and third periods. On the other hand, no differences may have been observed between the first two studies (IPD1 and IPD2) because the second study (IPD2) began less than 2 years after PCV7 introduction in Peru, before sufficient vaccine coverage could impact pneumococcal serotype diversity. Rodriguez-Ruiz *et al*. proposed that in the UK, changes in serotype diversity are primarily associated with vaccine introduction rather than antibiotic use [27]. However, we consider that, in addition to the selective pressure carried out by PCVs, which modulates serotype diversity and distribution, antibiotic resistance plays an important role. As hypothesized in our previous study, the increase in macrolide resistance could be directly associated with the increase of serotype 19A-ST320, which has been reported as a vaccine escape carrying the transposon Tn2010 that confers resistance to macrolides [28,30]. This implies that the observed reduction in pneumococcal serotype diversity may result not only from the direct effects of PCV10 and PCV13 but also from antibiotic resistance, particularly in Peru, where macrolides are sold over the counter and are commonly used as empirical therapy for respiratory infections, second only to penicillin [31].

WGS-predicted susceptibility showed a high correlation (>90%) with MIC for penicillin and macrolides. This is similar to the correlation found for penicillin and erythromycin in isolates from children with IPD in Argentina [15] and the US [32]. The prediction of antimicrobial resistance by WGS appears to be comparable to MIC interpretation, demonstrating its potential as a tool for antimicrobial resistance surveillance when strain isolation is not feasible. However, MIC remains the gold standard for determining antimicrobial resistance. Additionally, we suggest performing a detailed evaluation of mobile genetic elements, such as the Macrolide Efflux Genetic Assembly (mega) element, which carries macrolide efflux and ribosomal protection and is often linked to specific serotypes and/or STs [29,30, 33].

In our study, only one very major discrepancy (resistant by phenotypic methods but susceptible as predicted by WGS) was found in an isolate for penicillin and ceftriaxone, five for macrolides and seven for tetracycline. We also found a low overall concordance for ceftriaxone (70.1%) and tetracycline (78.1%). According to the GPS pipeline, such discrepancies may occur because the system cannot assign the intermediate category for some antibiotics, as no known genes or mutations currently explain intermediate resistance for some of them [12]. Therefore, it is difficult to assess the acceptability of these concordance levels, since the clinical impact of a resistant isolate being reported as susceptible is not equivalent to that of a susceptible or intermediate isolate being reported as resistant.

This study has some limitations. First, the strains analysed were obtained through passive surveillance studies conducted in hospitals in Lima and therefore may not accurately reflect the overall distribution of pneumococcal strains in Peru. Second, the data reflect proportional changes in the distribution of serotypes; however, true changes in the incidence of individual serotypes could not be assessed due to the unavailability of disease burden data. Nevertheless, this study has some important strengths. The primary strength is the integration of WGS into our pneumococcal surveillance studies, allowing the detection of changes beyond serotype alone. This allows for the identification of pneumococcal lineages driving serotype replacement and shifts in antibiotic resistance following the introduction of PCVs. Additionally, the study highlights the reliability of genomic data for accurately inferring both serotype and antibiotic resistance among isolates.

In summary, this study, which outlines changes in the epidemiology of pneumococcal disease in Lima, Peru, demonstrates that the pneumococcal population continues to evolve in the context of vaccine introduction. Future studies should monitor changes in 19A and other emerging serotypes in Peru and the region. Therefore, ongoing surveillance in the post-PCV15/PCV20 era is essential to guide future vaccine development, implementation and empiric antibiotic treatment.

## Supplementary material

10.1099/mgen.0.001621 Uncited Supplementary Material 1.

10.1099/mgen.0.001621 Uncited Supplementary Material 2.

## Abbreviations

AMR: antimicrobial resistance
CC: clonal complex
CDC: centers for disease control and prevention
GPIN: Peruvian Pneumococcus Research Group/Grupo Peruano de Investigación en Neumococo
GPS: Global Pneumococcal Sequencing Project
GPSC: Global Pneumococcal Sequence Cluster
IPD: invasive pneumococcal disease
KB: Kirby-Bauer method
MIC: minimum inhibitory concentration
MLST: multilocus sequence typing
NS: non-susceptibility
PBPs: penicillin-binding proteins
PCV: pneumococcal conjugate vaccine
PCV7: 7-valent pneumococcal conjugate vaccine
PI-1: pathogenicity islet 1
ST: sequence type
WGS: whole-genome sequencing
