# Supplementary material for: Genomic features of pneumococcal strains isolated from paediatric patients with invasive disease during pneumococcal conjugate vaccine introduction in Lima, Peru
Source: Microb Genom. 2026 Feb 3;12(2):001621. doi: 10.1099/mgen.0.001621 (PMC12868906; doi:10.1099/mgen.0.001621)
Supplement: Uncited Supplementary Material 2. [file mgen-12-01621-s002.pdf]

Supplementary material 1: Comparison of penicillin MICs predicted by whole genome sequence pipeline penicillin-binding protein types and MIC determination by E-test in IPD strains (N=241)

Phenotype MIC (n)	Predicted MIC (n)	Change in the interpretation	PBP1A / PBP2B / PBP2X (n)	Serotype/ST/GPSC (n)
R<br>1 (1)	S<br>0.03 (1)	1	2/0/2 (1)	7F/191/15 (1)
S<br>0.008 (1)<br>0.016 (1)<br>0.023 (1)<br>0.064 (3)<br>0.075 (1)	R<br>0.12 (2)<br>0.25 (2)<br>1 (2)<br>2 (1)	7	19/31/182 (2)<br>0/1/1 (1)<br>2/12/108 (1)<br>2/53/77 (1)<br>7/1/300 (1)<br>13/16/183 (1)	6C/1292/89 (2)<br>6B/315/47 (1)<br>14/9054/18 (1)<br>16F/7438/386 (1)<br>19F/newST/new (1)<br>24F/338/5 (1)

IPD: Invasive pneumococcal disease.
[a] Meningitis breakpoint: S: MIC ≤ 0.06 ug/ml, R: MIC ≥ 0.12ug/ml ST: Sequence type.
ST: Sequence type.
GPSC: Global Pneumococcal Sequencing Custer.
newST: new sequence type.
new: new GPS cluster.

Supplementary material 2: Comparison of ceftriaxone MICs predicted by whole genome sequence and MIC determined by E-test in IPD strains (N=241)

Phenotype MIC (n)	Predicted MIC (n)	Change in the interpretation	Serotype/ST/GPSC (n)
S<br>0.016 (1)<br>0.023 (1)<br>0.19 (2)<br>0.38 (3)<br>0.5 (26)	I<br>1 (33)	33	14/156/6 (18)<br>6B/90/23 (2)<br>19F/1421/1 (2)<br>23F/81/16 (2)<br>6B/902/321 (1)<br>6B/1624/23 (1)<br>14/5458/new (1)<br>14/9912/6 (1)<br>19A/320/1 (1)<br>19A/newST/59 (1)<br>19F/9904/16 (1)<br>19F/newST/new (1)<br>23F/156/6 (1)
S<br>0.032 (1)<br>0.38 (2)<br>0.5 (7)	R<br>2 (10)	10	19A/320/1 (10)
I<br>0.75 (9)<br>1 (11)	R<br>2 (20)	20	19A/320/1 (19)<br>19A/1451/1 (1)
I<br>0.75 (3)<br>1 (1)	S<br>0.5 (4)	4	7F/191/15 (1)<br>23B/6149/5 (1)<br>24F/230/10 (1)<br>24F/newST/10 (1)
R<br>1.5 (2)<br>2 (1)<br>4 (1)	I<br>1 (4)	4	14/646/44 (1)<br>19A/276/10 (1)<br>19F/5459/1 (1)<br>19F/1421/132 (1)
R<br>3 (1)	S<br>0.5 (1)	1	10A/5472/266 (1)

IPD: Invasive pneumococcal disease.
[a] Meningitis breakpoint: S: MIC ≤ 0.5ug/ml, I: MIC=1ug/ml, R: MIC ≥ 2ug/ml.
ST: Sequence type.
GPSC: Global Pneumococcal Sequencing Custer.

Supplementary material 3: Comparison of macrolide-resistance predicted by whole genome sequence and MIC determined by E-test in IPD strains (N=241)

Phenotype MIC (n)	Predicted MIC (n)	Change in the interpretation	*emr*B/*mef*A/*msr*D (n)	Serotype/ST/GPSC (n)
I<br>0.5 (1)<br>0.64 (1)<br>1 (3)<br>0.75 (3)	S<br>0.06 (8)	8	neg/neg/neg (8)	3/180/12 (1)<br>6A/1876/13 (1)<br>6B/5625/105 (1)<br>7F/191/15 (1)<br>10A/18188/266 (1)<br>23B/1349/5 (1)<br>23F/156/6 (1)<br>33F/1012/3 (1)
R<br>1 (2)<br>1.5 (2)<br>256 (1)	S<br>0.06 (5)	5	neg/neg/neg (5)	6B/1121/23 (1)<br>14/156/6 (1)<br>15B/3557/48 (1)<br>23B/1349/5 (1)<br>35B/18270/72 (1)
S<br>0.016 (1)<br>0.094 (5)	R<br>8 (1)<br>32 (5)	6	pos/pos/pos (3)<br>pos/neg/neg (2)<br>neg/pos/pos (1)	14/156/6 (2)<br>6C/1292/89 (1)<br>18F/5456/9 (1)<br>19A/320/1 (1)<br>19A/newST/1 (1)

IPD: Invasive pneumococcal disease.
ST: Sequence type.
GPSC: Global Pneumococcal Sequencing Custer.
newST: new sequence type.
Neg: gene absent; Pos: gene present.

Supplementary material 4: Comparison of tetracycline-resistance predicted by whole genome sequence and Kirby Bauer (KB) testing in IPD strains (N=247)

KB breakpoint (n)	Tetracycline-resistance predicted (n)	Change in the interpretation	*tet* gene (n)	Serotype/ST/GPSC (n)
I (32)	S (32)	32	neg (32)	14/156/6 (6)<br>10A/5472/266 (3)<br>5/289/8 (2)<br>6B/5449/105 (2)<br>23A/439/7 (2)<br>Others 17 patters
I (10)	R (10)	10	*tet*M (8)<br>neg (2)	14/156/6 (3)<br>19A/320/1 (2)<br>14/646/44 (1)<br>19F/1421/1 (1)<br>23F/81/16 (2)<br>24F/230/10 (1)
R (7)	S (7)	7	neg (7)	14/156/6(2)<br>6B/1121/23(1)<br>6B/5625/105(1)<br>11A/193/11 (1)<br>19A/5452/69 (1)<br>34/5447/45 (1)
S (5)	R (5)	5	*tet*M (4)<br>neg (1)	6C/1292/89 (1)<br>23F/242/14 (1)<br>19F/1421/132 (1)<br>19F/newST/new (1)<br>24F/4253/10 (1)

IPD: Invasive pneumococcal disease.
ST: Sequence type.
GPSC: Global Pneumococcal Sequencing Custer.
newST: new sequence type.
new: new GPS cluster.
Neg: gene absent.
